# Malignancy Risk Analysis in Patients with Inadequate Fine Needle Aspiration Cytology (FNAC) of the Thyroid

**DOI:** 10.1371/journal.pone.0049078

**Published:** 2012-11-19

**Authors:** Talib Al Maqbali, Miroslav Tedla, Martin O. Weickert, Hisham Mehanna

**Affiliations:** 1 Institute of Head & Neck and Education Studies, University Hospital Coventry and Warwickshire, Coventry, United Kingdom; 2 Warwickshire Institute for the Study of Diabetes, Endocrinology and Metabolism, University Hospitals, Coventry, United Kingdom; 3 Clinical Sciences Research Institute, Warwick Medical School, University of Warwick, Coventry, United Kingdom; 4 Institute of Head and Neck Studies and Education (InHANSE), Divisional Offices, University Hospital, Coventry, United Kingdom; Consiglio Nazionale delle Ricerche (CNR), Italy

## Abstract

**Background:**

Thyroid fine needle aspiration cytology (FNAC) is the standard diagnostic modality for thyroid nodules. However, it has limitations among which is the incidence of non-diagnostic results (Thy1). Management of cases with repeatedly non-diagnostic FNAC ranges from simple observation to surgical intervention. We aim to evaluate the incidence of malignancy in non-diagnostic FNAC, and the success rate of repeated FNAC. We also aim to evaluate risk factors for malignancy in patients with non-diagnostic FNAC.

**Materials and Methods:**

Retrospective analyses of consecutive cases with thyroid non diagnostic FNAC results were included.

**Results:**

Out of total 1657 thyroid FNAC done during the study period, there were 264 (15.9%) non-diagnostic FNAC on the first attempt. On repeating those, the rate of a non-diagnostic result on second FNAC was 61.8% and on third FNAC was 47.2%. The overall malignancy rate in Thy1 FNAC was 4.5% (42% papillary, 42% follicular and 8% anaplastic), and the yield of malignancy decreased considerably with successive non-diagnostic FNAC. Ultrasound guidance by an experienced head neck radiologist produced the lowest non-diagnostic rate (38%) on repetition compared to US guidance by a generalist radiologist (65%) and by non US guidance (90%).

**Conclusions:**

There is a low risk of malignancy in patients with a non-diagnostic FNAC result, commensurate to the risk of any nodule. The yield of malignancy decreased considerably with successive non-diagnostic FNAC.

## Introduction

Thyroid nodules are common in clinical practice. Using ultrasound scanning, the prevalence of thyroid nodules can reach up to 50% of the population [1]. Approximately 5% of these nodules have been shown to be malignant [2]. Fine needle aspiration cytology (FNAC) is the accepted standard tool for the evaluation of thyroid nodules [3]–[12]. It is safe and accurate with reported high sensitivity and specificity for malignancy [13], [14]. It is also reported to reduce the need for thyroid surgery by half [15] and to reduce the overall financial costs of medical care by 25% [2].

However, FNAC does have limitations, which include a significant rate of non-diagnostic results. This ranges from 0.6% [16] to 43.1% [17]. Nomenclature for inadequate FNAC varies in the literature causing unnecessary confusion [18]. It includes “inadequate”, “unsatisfactory”, “non diagnostic” and/or ‘Thy1’ (Thy1 category according to British Thyroid Association classification system). In this manuscript, we use the term non-diagnostic. The management strategies for these patients range in the literature from simple observation, to ultrasound surveillance to surgical intervention [19]. The recommended approach by both the British Thyroid Association and the American Thyroid Association is to repeat the biopsy [20]–[23]. However, repeating the biopsy may not always result in a definitive diagnosis, even if the procedure is done under ultrasound guidance. In addition, repeating the biopsy carries financial implications [24] and may not be acceptable to patients [25].

In this study, we aimed to determine the malignancy rate in cases where the FNAC result was non-diagnostic (Thy1), and to determine the success rates of successive FNAC in achieving a definitive cytology diagnosis in the setting of an initial non-diagnostic result. In addition, we aimed to identify risk factors that are associated with malignancy in nodules with a non-diagnostic (Thy1) presentation.

## Materials and Methods

This was retrospective clinical audit from patient's medical records. The research was limited to secondary use of information previously collected in the course of normal care and data were anonymised before the conduction of statistical analyses. Therefore, this research did not fulfil the requirements for Research Ethics Committee (REC) review. This is in accordance with the Governance Arrangements for Research Ethics Committees (GAfREC) published by the UK Health Department in May 2011 (http://nres.nhs.uk/applications/approvalrequirements/ethical-review-requirements). Retrospective analyses were performed on all consecutive cases with a thyroid FNAC report of non-diagnostic (Thy1) undertaken in a tertiary care centre (University Hospital Coventry and Warwickshire, UHCW) between March 2005 and September 2010. Cases were identified by a search of the Hospital's cyto-pathological database which prospectively documents the patient details, the site of FNAC and the result. Our institution protocol stipulates that if the first FNAC is reported as non-diagnostic (Thy1), then the patient is normally advised to have a repeat FNAC. However if there was strong suspicion of malignancy or the patient declined further biopsy, the patient would be offered surgery at that point. If the FNAC did not yield a diagnosis after a second FNA, then the patient is usually advised to undergo surgery. If the patients decline surgery, they usually are then offered a further FNAC.

The FNAC procedure was carried out using a standard 21 gauge needle. Sampling typically targets the solid component of the lesion if present. If there is more than one nodule, a sample was taken from any nodule with suspicious or atypical ultrasound characteristics.

### Slides processing, reading and labelling

After the samples were taken, a preservative medium was added and the mixture was centrifuged. Samples were then analysed using liquid based cytology techniques. This was followed by the staining process, where two slides are prepared with Papanicolaou's (Pap) and May-Grunwald Giemsa (MGG) stains. Results are reported using the British Thyroid Association guidelines [1]. Specimens are considered adequate if they contain six or more groups of over 10 thyroid follicular cells, but the balance between cellularity and colloid is more important [1]. The FNAC was categorised as non-diagnostic Thy1 when samples do not meet the aforementioned criteria or where technical artefact precludes interpretation. The final histopathology diagnosis of the surgical specimen was based on WHO criteria.

### Data extraction and analysis

Patient's demographics, biochemistry, cytology, histopathology results and patient's letters were available on computerised patient records system. Lesion characteristics such as number, size, consistency and suspicious features on U/S were extracted from electronic ultrasound reports. SPSS software (version 18.0, Chicago Illinois) was used to perform the statistical analysis. Patient characteristics are presented as mean +/− SE. Univariate analysis of lesions characteristics were used to calculate the risk and odds ratio with confidence intervals. Statistical significance was defined as p<0.05.

## Results

**Figure 1:**
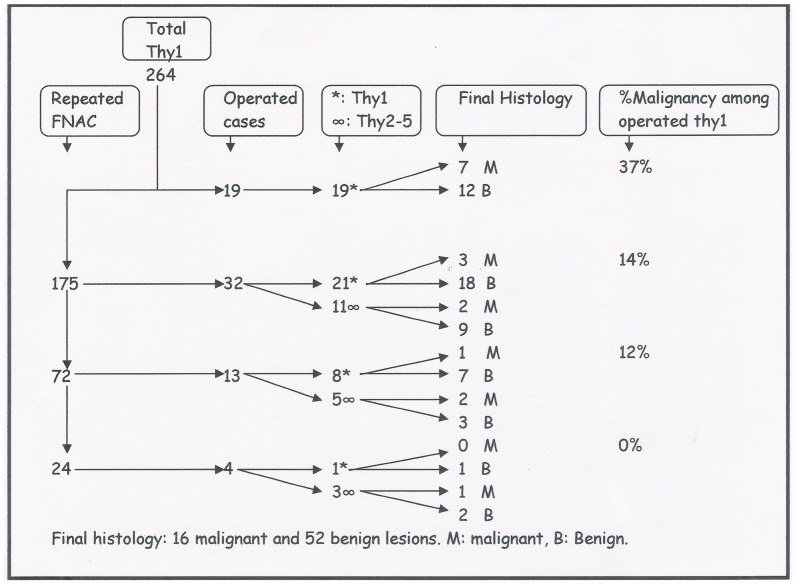
Flow chart showing details of the thyroid non diagnostic FNAC and their cytological and histological correlation.

1657 FNAC were performed during the period of the study. A total of 452 were reported as non-diagnostic (27.3%). 264 out of 452 samples were non-diagnostic on first FNAC and constitute the population of our study. The age of patients ranged from 16 to 90 years with mean age of 54.02±1.03 years and median age of 54 years. There were 212 females (80.3%), and 52 males (19.7%). Please refer to Figure 1 for further details.

### Malignancy rate in non-diagnostic cases

In total, 16 (6%) of thy1 cases were diagnosed as having thyroid cancer on histology. Four patients had incidental papillary thyroid microcarcinoma in addition to a benign thyroid lesion (see table 1). These four cases were not included in the analysis for risk of malignancy as they were incidental findings and were not found in the sampled nodule. Therefore, the overall malignancy rate was 4.5% (12/264). The malignancy rate among operated cases was 18% (12/68).

**Table 1:** Showing details of final histopathology results for the 68 operated cases.

Benign final histology	n	Malignant final histology	n
Hyperplastic/degenerative nodule	21	Papillary thyroid carcinoma	5
Adenoma	15	Minimally invasive follicular cancer	3
Multinodular goiter	15	Hurthle cell carcinoma	2
Thyroiditis and others	5	Anaplastic carcinoma	1
		Non Hodgkin's Lymphoma	1
Total	56		12

NB: 4 cases with incidental papillary thyroid microcarcinoma are listed in the benign category in this table.

Of the 264 patients with non diagnostic (Thy1) results on first FNAC, only 7% (19 patients) were operated on without a repeat FNAC. Most of these patients were operated on due to clinical suspicion and a high malignancy rate 37% (6/19) was found in this group. Malignancy rates after having successive non-diagnostic FNAC was much lower: 14% (5/21) after second non-diagnostic FNAC and 0% after fourth FNAC. In total, out of the 264 cases with Thy1 result in initial FNAC, only 10% (27/264) had more than three FNAC during their spell of care.

### Effect of ultrasound guidance on repetition of non-diagnostic FNAC

79% of the first 264 FNAC were done using ultrasound guidance. This increased to 93% and 96% on second and the third FNAC respectively. Overall, repeating the FNAC on these lesions using palpation resulted in 90% non-diagnostic rate compared to 65% and 38% non-diagnostic rates when FNAC were repeated by a general radiologist and specialist radiologists respectively, P = 0.001. Please refer to table 2 for details.

**Table 2:** Portions of FNAC done under ultrasound guidance and results of repeating FNAC.

	Number of FNAC	US guided	Non-diagnostic Thy1
1^st^ FNAC	264	79%	264
2^nd^ FNAC	175	93%	108 (62%)
3^rd^ FNAC	72	96%	34 (47%)
4^th^ FNAC	24	87%	12 (50%)
5^th^ FNAC	2	50%	2 (100%)
6^th^ FNAC	1		0

### Risk factors for malignancy in non-diagnostic FNACs

We performed a univariate regression analysis of known malignancy risk features including age, gender, number of nodules, size of the lesion on ultrasound, size of the lesion on pathology examination and nodule consistency. Only number of nodules were found to be prognostic of malignancy with cancers being more common in cases with multiple nodules (P = 0.006), see table 3.

**Table 3:** Risk factor analysis for malignancy in inadequate FNAC.

	Odds ratio	95% CI for odds ratio	P value
HPE size (≥4 cm VS <4 cm)	3.4	0.865–13.492	0.080
Gender (male VS female)	3.28	0.864–12.497	0.081
Consistency (solid VS cystic)	2.75	0.458–16.525	0.269
Age (≥50years VS <50 years)	2	0.540–7.409	0.300
US size (≥4 cm VS <4 cm)	1.64	0.457–5.94	0.45
TSH (<0.36 VS 0.36–6.0)	1.02	0.191–5.473	0.98
No of nodules (multiple vs single)			0.006

## Discussion

Our study demonstrates several important findings. First, the yield of malignancy decreased considerably with successive non-diagnostic FNAC, whilst the proportion of operated patients increased. We found that if the result was non-diagnostic for the second time, the risk of malignancy decrease by two thirds, and after three inadequate FNAC the risk was nil in our cohort. This confirm findings by other reports for example, Renshow [26] found that patients with at least two non diagnostic FNAC had significantly lower risk of malignancy (0%) compared to those who had only one non diagnostic FNAC (20%). On the other hand, Jo et al [27] found that there is no relation between the malignancy rate and the number of non diagnostic aspirations. We believe that the high malignancy yield in patients with one non diagnostic FNAC is rational due to the presence of other clinical and sonographic suspicious features for which those patients were operated. Therefore, the increased malignancy risk is not due to the fact they had one non diagnostic FNAC but it is due to the presence of other suspicious malignancy characteristics. Our study showed overall 4.5% malignancy risk for nodules with non diagnostic FNAC. This is comparable to malignancy risk of any nodule.

The second observation is that the repetition of FNAC yields a diagnostic cytology result in about 45% of the cases, with most of the cases having up to two repeated FNAC and few having more than three FNAC.

Third, repetition under ultrasound guidance by a head and neck radiologist appeared to be the most effective method, compared to a general radiologist or by palpation. Fourth, multiple nodules on ultrasound appeared to be a prognostic factor for malignancy in patients with non-diagnotic (Thy1) FNAC results.

### Significance of non diagnostic FNAC

There has been debate over the significance of non diagnostic FNAC. The overall rate of malignancy in thyroid non diagnostic FNAC ranges between 1.7% [28] to 11.3% [29] in the literature (when cases of incidental papillary thyroid microcarcinoma are excluded) (see table 4). We report a rate of 4.5% histologically confirmed malignant results, with comparable rates reported in other studies [19], [30]–[33]. The malignancy rate would be higher (6%) if incidental papillary thyroid microcarcinomas are included. We believe that incidental papillary microcarcinoma detected on post operative histology should be excluded as these lesions are not detected or sampled pre operatively [34]. The inclusion of these tumours may introduce bias to the results. For example, Oertel et al [32] reported a 3.4% overall rate of malignancy in non-diagnostic FNAC. This rate increased to 11% if incidental papillary thyroid microcarcinomas were included. Therefore, it is important that authors document this when reporting and calculating the overall rate of malignancy.

**Table 4:** Non diagnostic and malignancy rate for thyroid FNAC in the literature.

Author	year	Total FNAC done	ND Rate	patients with ND FNAC	Number operated patients	Malignancy among ND	Malignancy among operated cases
Yoon et al [37]	2010	22754	16.3%	NA	230	2.7%	43.9%
Gharib and Goellner [38]	1993	18183	15%	NA	NA	NA	NA
Piana et al [28]	2010	18000	12.5%	1342	96	1.7%	24%
Chow et al [19]	2001	17887	21%	150	27	7%	37%
Oertei et al [32]	2007	9397	1%	117	38	3.4%	11.3%
Caruso,and Mazzaferri [5]	1991	9119	22%	NA	NA	NA	NA
Slowinaka et al [25]	2004	4601	8.9%	408	NA	6.6%	NA
Baloch et al [29]	2003	3007	8%	237	53	11.3%	51%
Baier et al [39]	2009	944	11.8%	NA	NA	NA	NA
Deandrea et al [35]	2010	927	NA	NA	51	NA	5.8%
Redman et al [40]	2006	693	4%	NA	NA	NA	NA
Baloch et al [33]	1998	662	11%	72	8	2.7%	25%
Bellantone et al [41]	2004	575	9.2%	NA	NA	NA	NA
Ceresini et al [16]	2004	465	0.6%	307	NA	NA	NA
Cai et al [42]	2006	434	7.3%	NA	NA	NA	NA
Singh et al [43]	2003	423	25%	NA	NA	NA	NA
Schmidt et al [30]	1997	345	17.1%	59	21	2%	NA
Tabaqchali et al [17]	2000	239	43.1%	77	NA	3.9%	5.2
Bakshi et al [34]	2003	128	35%	45	45	NA	2.2%
Macdonald and Yazdi [31]	1996	NA	NA	91	NA	2%	NA
McHenry et al [23]	1993	NA	NA	92	NA	NA	9%

ND: non diagnostic FNAC.

The rate of malignancy found in operated cases with non-diagnostic FNAC varies widely between studies, ranging from 2.2% to 51% [17], [19], [23], [28], [29], [32]–[35] (see table 4).A high rate of malignancy among operated cases for non diagnostic FNAC may be due to patient selection bias i.e. those patients with a non-diagnostic (Thy 1) result who are suspected of having malignancy are more likely to undergo surgery [19], [36]. Therefore, consideration of the overall rate of malignancy is probably more important in determining our treatment strategy towards non-diagnostic FNAC patients.

Our study also demonstrates that the risk of malignancy declines with each successive repetition of non-diagnostic FNA, Patients who had more than 3 non-diagnostic FNAs in our series had no malignancies detected. This would suggest a possible role for observation in patients who have had three or more non-diagnostic FNAC results and there is no clinical suspicion of malignancy. However, this finding would need to be independently validated in other cohorts before a change of practice may be contemplated, within the context of a study.

There is some debate regarding the need for ultrasound-guidance during needle aspiration, and whether it decreases the non-diagnostic rate [44]. Some studies have shown that ultrasound guidance does improve the hit rate and the accuracy of FNAC [45]–[46]. Our results demonstrate that in the context of a non-diagnostic initial FNAC, ultrasound guidance, especially by an experienced head and neck radiologist resulted in a considerable improvement in the non-diagnostic rate. The fact that the non-diagnostic rate in the second and third FNACs was considerably higher than on initial FNAC suggests that this subset of lesions are difficult to obtain definitive cytological results on. This may be partly due to the fact that some are cysts with little or no solid or cellular content.

The rate of non diagnostic FNAC has a very wide range in literature (table 4). The reasons for this are variable and beyond the scope of this paper, however, we reported the results of FNAC prepared using liquid base cytology which is reported to have less non diagnostic rate compared to conventional cytology results [47], [48].

### Risk factors for malignancy in non-diagnostic FNAC

In the literature, few studies have addressed possible risk factors specific for malignancy in non-diagnostic FNAC. For example, McHenry et al [23] identified male gender as a possible predictor of malignancy (P<0.05) in nodules with non-diagnostic FNAC. In contrast, Mendelson et al [49] found that male gender is not associated with high risk of malignancy and they did not find any statistically significant risk associated with radiation exposure, family history of malignancy, solitary nodule or nodule more than 3 cm. Similarly, Chow et al [19] reported no significant correlation between pre-operative findings and risk of malignancy including the number of nodules and ultrasound characteristics as well as physical findings. Our study identified multiple nodules on ultrasound as a significant risk factor for malignancy. In addition, it also demonstrated a possible trend for males to have a higher malignancy risk compared to females. Similarly, lesions sized ≥4 cm were more likely to be malignant compared to those less than 4 cm as were solid lesions compared to cystic lesions but again these associations did not reach statistical significance, table 3.

### Conclusions

Thyroid FNAC is the preferred diagnostic modality for the investigation of thyroid nodules. However, this method has limitations among which is the inadequacy rate. Our study showed that yield of malignancy in persistently non-diagnostic FNAC is low, and decreases with successive inadequate FNAC. Furthermore, ultrasound guidance, especially by an experienced head and neck specialist radiologist, improves the non-diagnostic rate.

## References

[1] British Thyroid Association (2007) Royal College of Physicians: British Thyroid Association Guidelines for the management of thyroid cancer. 2^nd^ edition. Internet: [http://www.british-thyroid-association.org/Guidelines/]. (cited 04/01/2011).

[2] Laszlo H (2004) The Thyroid Nodule. The new England Journal of Medicine 351: 1764–71. 15496625 10.1056/NEJMcp031436

[3] Pu RT, Yang J, Wasserman PG, Bhuiya T, Griffith KA, et al (2006) Does Hurthle cell lesion neoplasm predict malignancy more than follicular lesion neoplasm on thyroid fine-needle aspiration. Diagn Cytopathol 34 (5) 330–334. 16604553 10.1002/dc.20440

[4] Gharib H, Goellner JR (1993) Fine-needle aspiration biopsy of the thyroid: an appraisal. Ann Intern Med 15;118 (4) 282–9. 10.7326/0003-4819-118-4-199302150-00007 8420446

[5] Caruso D, Mazzafferri EL (1991) Fine needle aspiration biopsy in the management of thyroid nodules. Endocrinologist 1: 194–202.

[6] Mamoon N, Mushtaq S, Muzaffar M, Khan AH (1997) The use of fine needle aspiration biopsy in the management of thyroid disease. J Pak Med Assoc 47 (10) 255–258. 9529853

[7] Castro MR, Gharib H (2003) Thyroid fine-needle aspiration biopsy: progress, practice, and pitfalls. Endocr Pract 9 (2) 128–36. 12917075 10.4158/EP.9.2.128

[8] Cáp J, Ryska A, Rehorková P, Hovorková E, Kerekes Z, et al (1999) Sensitivity and specificity of the fine needle aspiration biopsy of the thyroid: clinical point of view. Clin Endocrinol (Oxf) 51 (4) 509–515. 10583320 10.1046/j.1365-2265.1999.00847.x

[9] Ko HM, Jhu IK, Yang SH, Lee JH, Nam JH, et al (2003) Clinicopathologic analysis of fine needle aspiration cytology of the thyroid. A review of 1,613 cases and correlation with histopathologic diagnoses. Acta Cytol 47 (5) 727–732. 14526669 10.1159/000326596

[10] Al-Hureibi KA, Al-Hureibi AA, Abdulmughni YA, Aulaqi SM, Salman MS, et al (2003) The diagnostic value of fine needle aspiration cytology in thyroid swellings in a university hospital, Yemen. Saudi Med J 24 (5) 499–503. 12847625

[11] Amrikachi M, Ramzy I, Rubenfeld S, Wheeler TM (2001) Accuracy of fine-needle aspiration of thyroid. Arch Pathol Lab Med 125 (4) 484–488. 11260620 10.5858/2001-125-0484-AOFNAO

[12] Schlinkert RT, Van Heerden JA, Schlinkert RT, Young WF Jr, Farley DR (1997) Factors that predict malignant thyroid lesions when fine-needle aspiration is “suspicious for follicular neoplasm.”. Mayo Clin Proc 72 (10) 913–916. 9379692 10.1016/S0025-6196(11)63360-0

[13] Gharib H, Goellner JR (1995) Fine-Needle Aspiration Biopsy Of Thyroid Nodules. Endocrine practice 1 (6) 410–417. 15251569 10.4158/EP.1.6.410

[14] Corrias A, Einaudi S, Chiorboli E, Weber G, Crinò A, et al (2001) Accuracy of Fine Needle Aspiration Biopsy of Thyroid Nodules in Detecting Malignancy in Childhood: Comparison with Conventional Clinical, Laboratory, and Imaging Approaches. The Journal of Clinical Endocrinology & Metabolism 86 (10) 4644–4648. 11600519 10.1210/jcem.86.10.7950

[15] Mazzaferri EL (1993) Management of a solitary thyroid nodule. New England Journal of Medicine 328 (8) 553–559. 8426623 10.1056/NEJM199302253280807

[16] Ceresini G, Corcione L, Morganti S, Milli B, Bertone L, et al (2004) Ultrasound-Guided Fine-Needle Capillary Biopsy of Thyroid Nodules, Coupled with On-Site Cytologic Review, Improves Results. Thyroid 14 (5) 385–389. 15186617 10.1089/105072504774193230

[17] Tabaqchali MA, Hanson JM, Johnsont SJ, Wadehrat V, Lennard TWJ, et al (2000) Thyroid aspiration cytology in Newcastle: a six year cytology/histology correlation study. Ann R Coll Surg Engl 82: 149–155. 10858674 PMC2503437

[18] Oertel YC (2006) Unsatisfactory (vs. nondiagnostic) thyroidal aspirates: a semantic issue? Diagnostic Cytopathology 34 (2) 87–88. 16511843 10.1002/dc.20460

[19] Chow LS, Gharib H, Goellner JR, Van Heerden JA (2001) Nondiagnostic thyroid fine-needle aspiration cytology: Management dilemmas. Thyroid 11 (12) 1147–1151. 12186502 10.1089/10507250152740993

[20] Orija IB, Hamrahian AH, Reddy SS (2004) Management of nondiagnostic thyroid fine-needle aspiration biopsy: survey of endocrinologists. Endocrine practice 10 (4) 317–323. 15760774 10.4158/EP.10.4.317

[21] Layfield LJ, Abrams J, Cochand-Priollet B, Evans D, Gharib H, et al (2008) Post-thyroid FNA testing and treatment options. A synopsis of the National Cancer Institute Thyroid Fine Needle Aspiration State of the Science Conference. Diagn Cytopathol 36: 442–448. 18478610 10.1002/dc.20832

[22] AACE/AME Task Force on Thyroid Nodules American Association of Clinical Endocrinologists and Associazione Medici Endocrinologi (2006) Medical guidelines for clinical practice for the diagnosis and management of thyroid nodules. Endocr Pract 12 (1) 63–102 Accessed online. Cited 10.02.2011. Available: http://www.scribd.com/doc/39020547/Thyroid-Guidelines. 10.4158/EP.12.1.63 16596732

[23] McHenry CR, Walfish PG, Rosen IB (1993) Non-diagnostic fine needle aspiration biopsy: A dilemma in management of nodular thyroid disease. The American Surgeon 59 (7) 415–419 (abstract only).. 8323073

[24] Borget I, Vielh P, Leboulleux S, Allyn M, Iacobelli S, et al (2008) Assessment of the cost of fine-needle aspiration cytology as a diagnostic tool in patients with thyroid nodules. Am J Clin Pathol 129: 763–771. 18426737 10.1309/H86KM785Q9KBWPW5

[25] Slowinska-Klencka D, Sporny D, Klencki M, Lewiński A (2004) Non-Diagnostic Cytological Outcome of Thyroid Biopsy and the Risk of Thyroid Malignancy. Endocrine pathology 15 (1) 65–75. 15067178 10.1385/ep:15:1:65

[26] Renshaw AA (2011) Significance of repeatedly nondiagnostic thyroid fine-needle aspirations. Am J Clin Pathol 135 (5) 750–2. 21502429 10.1309/AJCP8FX5CLPISSSK

[27] Jo VY, Vanderlaan PA, Marqusee E, Krane JF (2011) Repeatedly nondiagnostic thyroid fine-needle aspirations do not modify malignancy risk. Acta Cytol 55 (6) 539–43. 22156463 10.1159/000333230

[28] Piana S, Frasoldati A, Ferrari M, Valcavi R, Froio E, et al (2010) Is a five-category reporting scheme for thyroid fine needle aspiration cytology accurate? Experience of over 18 000 FNAs reported at the same institution during 1998–2007. Cytopathology DOI:10.1111/j.1365-2303.2010.00777.x. Cited 20.12.2010. 10.1111/j.1365-2303.2010.00777.x 20626438

[29] Baloch Z, LiVolsi VA, Jain P, Jain R, Aljada I, et al (2003) Role of repeat fine-needle aspiration biopsy (FNAB) in the management of thyroid nodules. Diagnostic Cytopathology 29 (4) 203–206. 14506672 10.1002/dc.10361

[30] Schmidt T, Riggs MW, Speights VO Jr (1997) Significance of nondiagnostic fine-needle aspiration of the thyroid. Southern Medical Journal 90: 1183–1186. 9404902 10.1097/00007611-199712000-00004

[31] MacDonald L, Yazdi HM (1996) Non diagnostic fine needle aspiration biopsy of the thyroid gland. Acta cytologica 40: 423–428. 8669173 10.1159/000333893

[32] Oertel Yolanda C, Miyahara-Felipe Leika, Mendoza Mayo G, Yu Kai (2007) Value of Repeated Fine Needle Aspirations of the Thyroid: An Analysis of Over Ten Thousand FNAs. Thyroid 17 (11) 1061–1066. 17910525 10.1089/thy.2007.0159

[33] Baloch W, Sack J, Yu H, Livolsi A, Gupta K (1998) Fine-Needle Aspiration of Thyroid: An Institutional Experience. Thyroid 8 (7) 565–569. 9709908 10.1089/thy.1998.8.565

[34] Bakshi A, Mansoor Ibrahim, Jones A (2003) Analysis of inconclusive fine needle aspiration of thyroid follicular lesions. Endocr Pathol 14 (2) 167–175. 12858008 10.1385/ep:14:2:167

[35] Deandrea M, Ragazzoni F, Motta M, Torchio B, Mormile A, et al (2010) Diagnostic Value of a Cytomorphological Subclassification of Follicular Patterned Thyroid Lesions: A Study of 927 Consecutive Cases with Histological Correlation. Thyroid 20 (10) 1077–1083. 20883171 10.1089/thy.2010.0015

[36] Kelman S, Rathan A, Leibowitz J, Burstein E, Haber S (2001) Thyroid cytology and the risk of malignancy in thyroid nodules: importance of nuclear atypia in indeterminate specimens. Thyroid 11 (3) 271–277. 11327619 10.1089/105072501750159714

[37] Yoon JH, Kwak JY, Kim EK, Moon HJ, Kim MJ, et al (2010) How to Approach Thyroid Nodules with Indeterminate Cytology. Annals of Surgical Oncology 17 (8) 2147–2155. 20217250 10.1245/s10434-010-0992-5

[38] Gharib H, Goellner JR (1993) Fine-needle aspiration biopsy of the thyroid: an appraisal. Ann Intern Med 118 (4) 282–9. 8420446 10.7326/0003-4819-118-4-199302150-00007

[39] Baier ND, Hahn PF, Gervais DA, Samir A, Halpern EF, et al (2009) Fine-needle aspiration biopsy of thyroid nodules: experience in a cohort of 944 patients. Am J Roentgenol 193 (4) 1175–9. 19770344 10.2214/AJR.08.1840

[40] Redman R, Zalaznick H, Mazzaferri EL, Massoll NA (2006) The Impact of Assessing Specimen Adequacy and Number of Needle Passes for Fine-Needle Aspiration Biopsy of Thyroid Nodules. Thyroid 16 (1) 55–60. 16487014 10.1089/thy.2006.16.55

[41] Bellantone R, Pio Lombardi C, Raffaelli M, Traini E, De Crea C, et al (2004) Management of Cystic or Predominantly Cystic Thyroid Nodules: The Role of Ultrasound-Guided Fine-Needle Aspiration Biopsy. Thyroid 14 (1) 43–47. 15009913 10.1089/105072504322783830

[42] Cai XJ, Valiyaparambath N, Nixon P, Waghorn A, Giles T, et al (2006) Ultrasound-guided fine needle aspiration cytology in the diagnosis and management of thyroid nodules. Cytopathology 17 (5) 251–256. 16961653 10.1111/j.1365-2303.2006.00397.x

[43] Singh N, Ryan D, Berney D, Calaminici M, Sheaff MT, et al (2003) Inadequate rates are lower when FNAC samples are taken by cytopathologists. Cytopathology 14 (6) 327–331. 14632730 10.1046/j.0956-5507.2003.00084.x

[44] Richards ML, Bohnenblust E, Sirinek K, Bingener J (2008) Nondiagnostic thyroid fine-needle aspiration biopsies are no longer a dilemma. American Journal of Surgery 196 (3) 398–402. 18558398 10.1016/j.amjsurg.2007.10.017

[45] Danese D, Sciacchitano S, Farsetti A, Andreoli M, Pontecorvi A (1998) Diagnostic accuracy of conventional versus sonography-guided fineneedle aspiration biopsy of thyroid nodule. Thyroid 8 (1) 15–21. 9492148 10.1089/thy.1998.8.15

[46] Carmeci C, Jeffrey RB, McDougall IR, Nowels KW, Weigel RJ (1998) Ultrasound-guided fine-needle aspiration biopsy of thyroid masses. Thyroid 8 (4) 283–9. 9588492 10.1089/thy.1998.8.283

[47] Saleh H, Bassily N, Hammoud MJ (2009) Utility of a liquid-based, monolayer. preparation in the evaluation of thyroid lesions by fine needle aspiration biopsy: comparison with the conventional smear method. Acta Cytol 53 (2) 130–6. 19365963 10.1159/000325113

[48] Rossi ED, Raffaelli M, Zannoni GF, Pontecorvi A, Mulè A, et al (2009) Diagnostic efficacy of conventional as compared to liquid-based cytology in thyroid lesions: evaluation of 10,360 fine needle aspiration cytology cases. Acta Cytol 53 (6) 659–66. 20014555 10.1159/000325407

[49] Mendelson AA, Tamilia M, Rivera J, Hier MP, Sherman M, et al (2009) Predictors of malignancy in preoperative nondiagnostic biopsies of the thyroid. Journal of Otolaryngology - Head and Neck Surgery 38 (3) 395–400. 19476774

